# Effects of a Mindfulness-Based Intervention on Self-Compassion and Psychological Health Among Young Adults With a History of Childhood Maltreatment

**DOI:** 10.3389/fpsyg.2019.02373

**Published:** 2019-10-23

**Authors:** Diane Joss, Alaptagin Khan, Sara W. Lazar, Martin H. Teicher

**Affiliations:** ^1^Developmental Biopsychiatry Research Program, McLean Hospital, Belmont, MA, United States; ^2^Department of Psychiatry, Massachusetts General Hospital, Boston, MA, United States; ^3^Department of Psychiatry, Harvard Medical School, Boston, MA, United States

**Keywords:** mindfulness, self-compassion, stress, childhood maltreatment, depression, anxiety

## Abstract

**Background:**

Individuals who were maltreated during childhood are faced with increased risks for developing various psychological symptoms that are particularly resistant to traditional treatments. This pilot study investigated the effects of a mindfulness based behavioral intervention for young adults with a childhood maltreatment history.

**Methods:**

This study looked at self-report psychological questionnaires from 20 subjects (5 males) before and after a mindfulness-based behavioral intervention, compared to 18 subjects (6 males) in the waiting list control group (age range 22–29); all subjects experienced mild-to-moderate childhood maltreatment. We analyzed changes in stress, anxiety, depression, mindfulness and self-compassion related to the intervention with linear mixed effects models; we also analyzed the relationships among questionnaire score changes with partial correlation analyses and mediation analysis.

**Results:**

Linear mixed effects model analyses revealed significant group by time interaction on stress (*p* < 0.01), anxiety (*p* < 0.05), and self-compassion (*p* < 0.01), with the mindfulness group having significant reduction in stress and anxiety (*p* < 0.01), and significant increase in mindfulness (*p* < 0.05) and self-compassion (*p* < 0.001). Partial correlation analyses showed that among all subjects from both groups, changes in mindfulness positively correlated with changes in self-compassion (*r* = 0.578, *p* = 0.001), which negatively correlated with changes in depression (*r* = −0.374, *p* = 0.05) and anxiety (*r* = −0.395, *p* < 0.05). Changes in self-compassion mediated, in part, the relationship between changes in mindfulness and changes in anxiety (average causal mediation effect = −4.721, *p* < 0.05). We observed a dose-dependent effect of the treatment, i.e., the number of intervention sessions attended were negatively correlated with changes in stress (*r* = −0.674, *p* < 0.01), anxiety (*r* = −0.580, *p* < 0.01), and depression (*r* = −0.544, *p* < 0.05), after controlling for the individual differences in childhood maltreatment severity.

**Conclusion:**

Our results suggest that, to some extent, the mindfulness-based intervention can be helpful for improving self-compassion and psychological health among young adults with a childhood maltreatment history.

**Clinical Trial Registration:**

www.ClinicalTrials.gov, identifier NCT02447744.

## Introduction

Childhood maltreatment is remarkably prevalent. “Child maltreatment is the abuse and neglect that occurs to children under 18 years of age. It includes all types of physical and/or emotional ill-treatment, sexual abuse, neglect, negligence and commercial or other exploitation, which results in actual or potential harm to the child’s health, survival, development or dignity in the context of a relationship of responsibility, trust or power. Exposure to intimate partner violence is also sometimes included as a form of child maltreatment” (World Health Organization, 2016). In the 1990s, a large scale survey, later known as the Adverse Childhood Experience study, was conducted on 17,337 adult HMO members at a preventative health clinic in California (Felitti et al., 1998). This study indicated that 63% of the participants had experienced at least one category of childhood maltreatment or household dysfunction, and over 20% experienced three or more categories of adverse childhood experience (Felitti et al., 1998). This study also found more adverse childhood experience was associated with increased risks for alcoholism, drug abuse, depression, suicide attempts, and other high risk health conditions (Felitti et al., 1998).

Childhood maltreatment is also associated with increased risks for multiple medical and psychiatric conditions throughout the entire lifespan (Pietrek et al., 2013). Childhood maltreatment also puts individuals at risk for developing borderline personality disorder (Herman et al., 1989) as well as other personality disorders (Johnson et al., 1999). Individuals with a childhood maltreatment history tend to have earlier onset of psychological symptoms, more episodes, more comorbid disorders (Bernet and Stein, 1999), as well as higher likelihood of resistance toward traditional treatments (Nanni et al., 2012).

In recent years, mindfulness-based or mindfulness-informed treatment programs showed promising effects for treating a wide variety of clinical symptoms and disorders, many of which are commonly experienced in individuals with a childhood maltreatment history. For example, mindfulness based cognitive therapy is a highly effective treatment for recurrent depression (Williams et al., 2014; Kuyken et al., 2016; Rodrigues et al., 2019), especially for patients with a childhood maltreatment history (Williams et al., 2014) dialectical behavioral therapy, which has a strong mindfulness element, is a highly effective treatment for borderline personality disorder (Linehan et al., 1999; Verheul et al., 2003; Soler et al., 2012; Moran et al., 2018) mindfulness-based interventions are also useful for treating anxiety disorders (Roemer and Orsillo, 2002; Evans et al., 2008; Goldin and Gross, 2010; McEvoy, 2019), and in particular have shown efficacy for treating symptoms of trauma (Follette et al., 2006; King et al., 2013; Banks et al., 2015). Given that individuals with a childhood maltreatment history typically have symptoms of multiple disorders and are resistant to traditional treatments, the present study aims to investigate the effect of a mindfulness based behavioral intervention program for reducing psychological symptoms in young adults with a history of childhood maltreatment, regardless of the primary psychopathology they currently present.

Mindfulness has been defined as paying attention in a particular way: on purpose, in the present moment, and non-judgmentally, in the service of self-understanding and wisdom (Kabat-Zinn, 1990). Because of the intrinsic “present-centered” nature of mindfulness practice, i.e., it encourages “non-judgment and acceptance of thoughts and emotions as they occur in the present moment” (Boyd et al., 2018, p. 7), its therapeutic value for people with traumatic memories has been recognized in recent years. Several mindfulness-based treatments have also been developed or adapted for treating Post-traumatic Stress Disorder (PTSD), “as an alternative technique for targeting symptoms of avoidance and negative cognitions, including self-blame, shame and guilt among individuals with PTSD” (Follette et al., 2006; Lang et al., 2012; Banks et al., 2015; Boyd et al., 2018, p. 8). Although not all individuals with a childhood maltreatment history demonstrate full scale PTSD per DSM standard, the “self-blame, shame and guilt” components of the symptomology are rather common among childhood maltreatment victims (Hoglund and Nicholas, 1995; Andrews, 1998; Stuewig and McCloskey, 2005; Dorahy and Clearwater, 2012). Therefore, we hypothesized that a mindfulness-based intervention could bring similar therapeutic benefits for individuals with a childhood maltreatment history.

Previous studies have also suggested that childhood maltreatment is associated with low “self-compassion” (Tanaka et al., 2011; Vettese et al., 2011), which has been associated with emotional dysregulation (Vettese et al., 2011), and higher risk of psychological distress and suicidality (Tanaka et al., 2011). Self-compassion “involves feelings of caring and kindness toward oneself in the face of personal suffering and involves the recognition that one’s suffering, failures and inadequacies are part of the human condition” (Neff, 2003, p. 224). The Self-Compassion Scale was developed to evaluate this construct with six subscales: self-kindness vs. self-judgment, which evaluates the tendency to be “gentle, supportive and understanding toward oneself” instead of “harshly judging oneself for personal shortcomings” (Neff, 2016, p. 265), common humanity vs. isolation, which evaluates the recognition for shared human experience in face of failure, mistakes and imperfection, and mindfulness vs. over-identification, which measures the ability to be aware of one’s present moment experience without being caught up with negative narratives (Neff, 2016).

A previous factor analysis found strong positive correlation between mindfulness and self-compassion (Baer et al., 2006), while several clinical trials indicate that participation in a mindfulness based intervention increased self-compassion (Birnie et al., 2010; Gard et al., 2012; Greenberg et al., 2018). Therefore, the present study also investigated the effect of the mindfulness-based intervention on self-compassion, and the possible role of self-compassion in mediating the therapeutic effects of the intervention.

## Materials and Methods

### Subject Enrollment

This study has been registered on clinicaltrials.gov, with a clinical trial identifier of NCT02447744; the detailed information of this clinical trial can be publicly accessed at https://clinicaltrials.gov/ct2/show/NCT02447744. This study was approved by the Institutional Review Board (IRB) (IRB#: 2014P000295) of *Partners HealthCare*, which is the IRB for Massachusetts General Hospital, McLean Hospital, and several other major hospitals in the Boston area.

Forty-three subjects were recruited from the subject pool of a previous study about childhood maltreatment (Khan et al., 2015). Subjects were enrolled into the study if they pass the following inclusion/exclusion criteria: (1) determined to have childhood maltreatment as having a score of at least one in the Adverse Childhood Experience questionnaire (Felitti et al., 1998) or at least one category of childhood maltreatment in the Maltreatment and Abuse Chronology of Exposure (MACE) questionnaire (Teicher and Parigger, 2015); (2) verified age between 21 and 35 years old; (3) no suicidal attempts during the past 6 months; (4) no history of neurological disorders or psychiatric disorders with psychotic features; (5) passing common MRI exclusion criteria; (6) no prior experience with the Mindfulness Based Stress Reduction (MBSR) program (Kabat-Zinn, 1990) or other systematic meditation programs; (7) committed to meeting the requirement of refraining from using illicit drugs throughout the course of the study; (8) provided written informed consent to participate in this study.

The whole trial included three waves of recruitment for three corresponding cohorts of the mindfulness-based intervention programs. Only two cohorts are included in the analysis of this study because the research component of the trial stopped abruptly while participants were still enrolled in the third cohort of intervention, due to reasons unrelated to the trial itself; as a result, there was no research data from the third cohort. During each cohort of recruitment, subjects were enrolled in the mindfulness-based intervention program if they were available to attend the majority of the 9 sessions of meetings in that cohort, and they were administered research questionnaires before the beginning and after the end of the intervention program; if they were not available to attend the majority of sessions on the schedule, they were placed on the waiting list for the intervention program of the next cohort, and they were administered research procedures first at the time of enrolment, then for a second time at least 2 months later, then a third time after they complete the intervention program. The majority of subjects on the waiting list dropped out from the study; only a total of six subjects completed the intervention program after their waiting period and provided research data for three time points, among them, only four subjects completed the research questionnaires analyzed in this paper.

### Subject Assessment and Research Procedures

Comprehensive clinical assessment about childhood experience and psychiatric history were completed in the previous study, the subject pool of which we recruited from Khan et al. (2015). When subjects were enrolled in the present study, clinicians administered the Longitudinal Interview Follow-up Evaluation - Psychiatric Status Ratings (LIFE) (Keller et al., 1987) to assess their current mental health condition to determine whether they meet the inclusion/exclusion criteria of the present study. During the structured interview when subjects were first recruited into the previous study (Khan et al., 2015), we collected demographic information such as subjects’ date of birth, ethnicity, biological sex, employment, and marital status. During the LIFE interview when subjected were recruited into this study, we re-evaluated their employment and marital status.

After subjects were consented and enrolled into the study, they completed the following psychological questionnaires online: Perceived Stress Scale (PSS; Cohen et al., 1994), State-Trait Anxiety Inventory-Trait subscale (STAI-t; Spielberger and Sydeman, 1994), Self-Compassion Scale (SCS; Neff, 2003), Beck Depression Inventory (BDI; Beck et al., 1996) and Mindful Attention Awareness Scale (MAAS; Brown and Ryan, 2003). Those online questionnaires were administered with the REDCap electronic data capture tools (Harris et al., 2009). Upon completion of the intervention program, subjects were administered the same questionnaires. Subjects on the waiting lists were administered the same questionnaires at the same time period as the subjects in the corresponding cohort of mindfulness-based intervention. Other research procedures were also administered at the same time, including additional questionnaires, MRI and an episodic memory test, and they will be reported in other publications. Examples of items on MAAS include: “I could be experiencing some emotion and not be conscious of it until some time later.”, “I break or spill things because of carelessness, not paying attention, or thinking of something else.”, and “I rush through activities without being really attentive to them.” Examples of items on SCS include: “(Self-Kindness) I try to be loving toward myself when I’m feeling emotional pain.”, “(Self-Judgment) I’m disapproving and judgmental about my own flaws and inadequacies.”, “(Mindfulness) When I’m feeling down I try to approach my feelings with curiosity and openness.”, “(Over-Identification) When I fail at something important to me I become consumed by feelings of inadequacy.”, “(Common-Humanity)When I’m down and out, I remind myself that there are lots of other people in the world feeling like I am.” and “(Isolation)When I’m feeling down, I tend to feel like most other people are probably happier than I am.”

### Mindfulness Based Intervention Program

The mindfulness-based intervention program lasted for eight continuous weeks, which included eight 2.5-hour long weekly meeting sessions that took place on a weekday evening plus one whole-day session on a weekend day. The mindfulness based intervention program was modeled after the Mindfulness-Based Stress Reduction (MBSR) program (Kabat-Zinn, 1990), with several modifications to adapt the program to our study population. The intervention program covered topics such as mindfulness and awareness, perception and perspectives, being present, responding vs. reacting to stress, stress coping strategies, dealing with difficult emotions, handling difficult communications, and using mindfulness in everyday life (Santorelli et al., 2017). Modification to the original MBSR program included: allowing subjects to choose the length of homework meditation practice as they see fit (2, 3, 10, 20 or 30 min, as opposed to the original 45 min); inclusion of the “3-minute breathing space” exercise which was originally a component of the mindfulness based cognitive therapy program and has been shown to be helpful for PTSD patients (King et al., 2013); and general emphasis on empowering the participants by giving them choices on participation (e.g., they could choose to speak or not during group discussions) and practices (e.g., they could choose the type of practice that work better for them during their homework practice, such as body scan meditation, breathing awareness meditation or yoga/mindful movement). Skills taught in the program included *body scan meditation* during which subjects slowly moved attention to each part of the body, *mindful yoga* which was a gentle form of yoga that emphasized awareness to body movements instead of the exercise aspect, *open awareness meditation* during which attention was on anything that came into awareness at each moment, and *breath awareness meditation* during which the focus of awareness was on breathing, as well as *loving-kindness meditation* during which mantra were repeated to send kind wishes to people of choice, e.g., “May you/I/my wife be happy, healthy and strong.” Subjects were instructed to keep a daily log of how many minutes they practiced each skill at home. Subjects on the waiting list were instructed to keep a daily log about their stress level and the things they did for stress reduction (e.g., hobbies).

### Data Analysis

**FIGURE 1:**
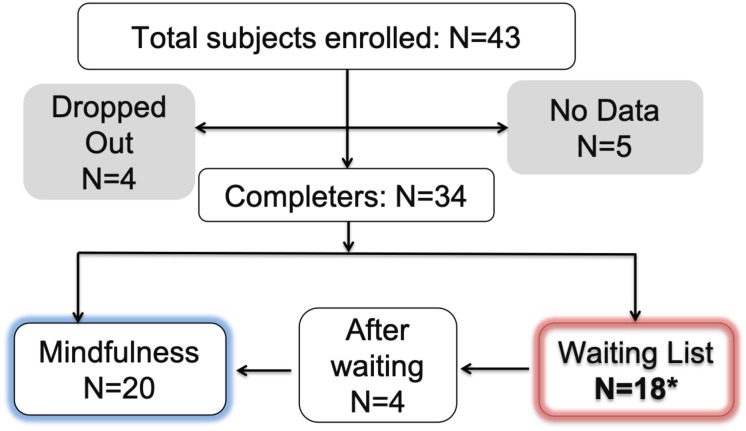
Flow chart of subject enrolment and retention.

A total of 34 subjects completed both testing time points or the two corresponding time points for the waiting list control. Among them, four subjects completed the mindfulness program after their waiting period, and thus contributed data from three time points: baseline, post-waiting period and post-intervention (Figure 1). Questionnaire data was extracted from the REDCap platform (Harris et al., 2009), and scored with scripts written in MATLAB^®^ R2012a (The MathWorks, Inc.).

Missing data was an issue for some research questionnaires, due to reasons such as subjects did not answer some items in a particular questionnaire or did not submit response to several questionnaires. If a subject did not answer some items in a certain questionnaire, or did not respond to the whole questionnaire, this subject’s data of this questionnaire at this particular assessment time point was discarded (i.e., marked as missing data) from further analysis. We reported the subject number (N) or degree of freedom (df) for each statistical analysis of each research questionnaire.

We first used ANCOVA, with group, age, sex, race and time-interval as covariates, to test whether the two groups had significant difference at baseline before the intervention. Then we used linear mixed effects models (Lindstrom and Bates, 1988) to evaluate the treatment effects on BDI, STAI-t, PSS, MAAS, and SCS. We used the implementation of this method in the “lme” function of the “nlme” package in R. The scores of each of the above-mentioned scales were used as the response/dependent variable for each model, with group (i.e., mindfulness or waiting list) and time point (i.e., before or after the intervention) as independent variables, and group by time interaction was the effect of interest in each model. In all models we used age, sex, ethnicity, and time-interval between the two measurements as covariates. Separate variance for each group and time point was used in all models. “REML” method was chosen in all models to maximize the restricted log-likelihood. We used the “r.squaredGLMM” function of the “MuMIn” package in R to estimate the percentage of variance explained in each linear mixed effects model. To obtain an effect size measure, we used the “ANOVA” function in R to compare each model with the alternative model without the factor of interest, which outputs the likelihood ratio and *p*-value as measurements of effect size. To test whether there was significant change after the intervention within the mindfulness group, we conducted further analysis with the “lmer” function in the “lme4” package of R, with age, sex, ethnicity and time-interval as covariates.

We used Pearson correlations to evaluate the relationships between variables of interest, and partial correlations to control for certain factors in the correlation analyses; in all correlation analyses we excluded cases with missing data in a “pairwise” manner.

Causal Mediation Analysis package in R (Tingley et al., 2014) was used to further investigate the relationships among score changes of research questionnaires. All subjects in both groups were included in the mediation analyses. The mediation analysis used score change of symptoms (BDI, STAI-t and PSS) as dependent variables, mindfulness (MAAS) score change as independent variable, with self-compassion (SCS) score change as the mediator.

## Results

### Demographics and Childhood Maltreatment Assessments

The two groups did not differ significantly in the distribution of sex [χ^2^(1, *N* = 38) = 0.320, *p* = 0.724) and race [χ^2^(4, *N* = 38) = 3.611, *p* = 0.579]. The two groups did not have any significant difference on employment status [χ^2^(4, *N* = 38) = 5.911, *p* = 0.206] or marital status [χ^2^(2, *N* = 38) = 0.266, *p* = 0.876]. The two groups had a marginally significant difference in age with the mindfulness group slightly older [*t*(36) = −1.755, *p* = 0.088], and a marginally significant difference in the interval between their two visits [*t*(36) = −1.764, *p* = 0.089] with the mindfulness group slightly longer due to the longer time interval of the four subjects from the waiting list. The two groups did not have significant difference in any of the assessments on childhood maltreatment or lifetime DSM diagnoses. Detailed information about subject demographics are listed in Table 1. Detailed childhood maltreatment assessment scores for each group are listed in Table 2. Percentages of DSM diagnoses in each group are listed in Table 3. Compliance of intervention session participation and homework practice in the mindfulness group is summarized in Table 4.

**TABLE 1:** Subject demographic information.

	**All subjects**	**Completers**	
		**Mindfulness**	**Waitlist**
Sample Size (N) [Female (F), Male (M)]	*N* = 43	*N* = 20	*N* = 18
	(F:31, M:12)	(F:15, M:5)	(F:12, M:6)
Age (in years) mean (SD, Range)	25.95	26.25	24.94
	(2.60, 22–34)	(2.099, 22–29)	(2.485, 22–29)
**Ethnicity (N)**			
White	28	12	13
Black/African American	6	5	1
Asian	4	2	2
Hispanic	4	1	1
Unknown	1	0	1
**Employment (N)^1^**			
Full time	27	15	10
Part time	5	4	2
Student	5	0	3
Unemployed	2	1	2
Volunteer	1	0	1
**Marital status (N)**			
Single	30	14	13
Married	6	4	4
Cohabiting for ≥1 year	4	2	1

*^1^The employment and marital status information for one subject in the mindfulness group and two subjects in the control group were from the original structured clinical interview when they were enrolled into the previous study due to missing data (Khan et al., 2015). Among subjects who did not complete the trial, there were three subjects for whom we did not have demographic information other than their age, sex, and ethnicity; and one subject we only had the employment and marital status information from the original interview (Khan et al., 2015).*

**TABLE 2:** Measurements of childhood maltreatment.

**Instruments**	**One-Way ANOVA**	**Mindfulness Group**		**Control Group**	
		**Mean (SE)**	**Range**	**Mean (SE)**	**Range**
ACE scores	*F*(1,35) = 0.007, *p* = 0.934	1.700 (0.398)	0–5	1.647 (0.507)	0–7
CTQ-emotional abuse	*F*(1,35) = 0.185, *p* = 0.669	9.250 (1.131)	5–21	9.941 (1.123)	5–21
CTQ-physical abuse	*F*(1,35) = 2.139, *p* = 0.153	6.900 (0.435)	5–11	6.000 (0.429)	5–11
CTQ-sexual abuse	*F*(1,35) = 0.282, *p* = 0.599	6.150 (0.998)	5–25	5.529 (0.471)	5–13
CTQ-emotional neglect	*F*(1,35) = 1.039, *p* = 0.315	9.350 (1.003)	5–18	11.059 (1.384)	5–22
CTQ-physical neglect	*F*(1,35) = 0.500, *p* = 0.484	6.200 (0.439)	5–10	6.706 (0.580)	5–13
MACE- number of types of maltreatment	*F*(1,35) = 0.168, *p* = 0.684	1.750 (0.481)	0–6	2.059 (0.591)	0–7
MACE-total severity scores of all types of maltreatment	*F*(1,35) = 0.254, *p* = 0.617	19.000 (3.294)	0–40	21.412 (3.448)	5–51
MACE-sexual abuse	*F*(1,35) = 0.000, *p* = 0.995	0.650 (0.274)	0–4	0.647 (0.363)	0–5
MACE-parental verbal abuse	*F*(1,35) = 0.056, *p* = 0.815	3.650 (0.862)	0–10	3.941 (0.872)	0–10
MACE-non-verbal emotional abuse	*F*(1,35) = 0.028, *p* = 0.869	2.800 (0.565)	0–8	2.941 (0.639)	0–8
MACE-parental physical maltreatment	*F*(1,35) = 0.766, *p* = 0.387	3.250 (0.512)	0–6	2.588 (0.556)	0–8
MACE-witnessing violence between parents	*F*(1,35) = 0.067, *p* = 0.797	1.000 (0.470)	0–8	1.176 (0.487)	0–6
MACE-witnessing siblings abused by parents	*F*(1,35) = 0.004, *p* = 0.948	0.850 (0.350)	0–5	0.882 (0.342)	0–3
MACE-peer verbal abuse	*F*(1,35) = 1.268, *p* = 0.268	4.300 (0.811)	0–10	5.647 (0.878)	0–10
MACE-peer physical abuse	*F*(1,35) = 0.585, *p* = 0.450	0.800 (0.360)	0–5	1.235 (0.450)	0–6
MACE-emotional neglect	*F*(1,35) = 0.642, *p* = 0.428	1.400 (0.358)	0–4	1.882 (0.499)	0–6
MACE-physical neglect	*F*(1,35) = 0.308, *p* = 0.583	0.300 (0.219)	0–4	0.471 (0.212)	0–2

**TABLE 3:** Lifetime DSM diagnoses percentages of both groups.

**DSM-IV-TR diagnosis**	**Frequencies**		**Fisher’s Exact Test**
	**Mindfulness**	**Control**	
Depressive disorders	7	5	*p* = 0.734
Anxiety disorders	8	7	*p* = 1
Personality disorders	1	3	*p* = 0.328
Major depressive disorder	3	4	*p* = 1
Bipolar disorder	0	0	N/A
PTSD	1	0	*p* = 1
Panic disorder	1	1	*p* = 1
Generalized anxiety disorder	5	3	*p* = 0.697
Social anxiety disorder	0	1	*p* = 0.474
Specific phobia	1	2	*p* = 0.595
Obsessive compulsive disorder	0	1	*p* = 0.474
Attention-deficit/hyperactivity disorder	3	1	*p* = 0.606
Substance use disorder	1	1	*p* = 1
Eating disorder	0	1	*p* = 0.474
Schizoid, dependent, obsessive compulsive or passive aggressive personality disorder	0	1	*p* = 0.474
Avoidant personality disorder	1	2	*p* = 0.595
Depressive, paranoid, schizotypal, histrionic, narcissistic, borderline or antisocial personality disorder	0	0	N/A

**TABLE 4:** Average number of class attended and minutes of homework practice in the mindfulness group.

	**Number of Classes**	**Homework Practice Skill Types (total minutes throughout the program)**				
		**Body Scan**	**Breath Awareness**	**Mindful Yoga**	**Open Awareness**	**Total**
Mean (SE)	7.250 (0.347)	174.850 (28.029)	179.550 (45.115)	255.150 (44.270)	108.600 (21.979)	718.150 (108.707)
Range	5–9	0–437	10–900	9–808	0–321	40–1804

### Questionnaire Results and Interaction Effects

ANCOVA analyses, with age, sex, race and time-interval as covariates, showed there were no group differences at baseline on any of the questionnaires (*p* > 0.25). Multivariate ANCOVA analyses, with group, age, sex, race and time-interval as covariates, showed no significant difference related to employment status [*F*(16,4) = 0.791, *p* = 0.676) or marital status [*F*(16,4) = 2.131, *p* = 0.243).

**FIGURE 2:**
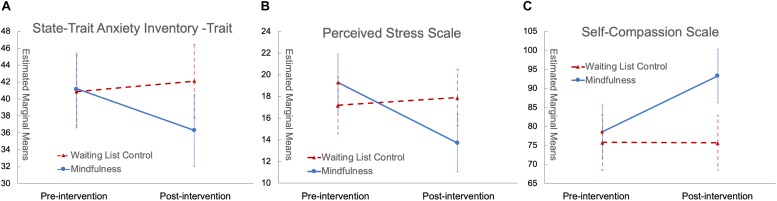
Significant group by time interaction with scores of STAI-t **(A)**, PSS **(B)**, and SCS **(C)**. In all panels, red triangles with dashed red lines represent the control group while blue circles with solid blue lines represent the mindfulness group; error bars are standard errors.

Fixed effects in the linear mixed effects model of STAI-t explained 19.49% of variance (Likelihood Ratio = 21.172, *p* = 0.032), and there was a significant group by time interaction effect [*F*(1,36) = 7.079, *p* = 0.012), with the mindfulness group having significant score reduction after the intervention [*F*(1,19) = 8.674, *p* = 0.008) while the waiting list control group had no significant change; the group by time interaction explained 2.92% of the variance in addition to the demographic covariates that explained 16.57% of the variance. The estimated marginal means for each group at each time point as well as their variance standard error (SE) are plotted in Figure 2A. To test whether history of childhood maltreatment was a moderator, we built another model to evaluate the 3-way interaction effect of “group” by “time” by “MACE-total severity scores of all types of maltreatment”. Fixed effects in this model explained 22.82% of variance (Likelihood Ratio = 30.562, *p* = 0.01), which was 3.33% more than the original model (Likelihood Ratio = 9.698, *p* = 0.046); there was a significant 3-way interaction [*F*(1,32) = 4.842, *p* = 0.035].

Fixed effects in the linear mixed effects model of PSS explained 16.08% of the variance (Likelihood Ratio = 22.160, *p* = 0.023), and there was a significant group by time interaction effect [*F*(1,36) = 9.486, *p* = 0.004] with the mindfulness group having significant score reduction after the intervention [*F*(1,18.35) = 12.934, *p* = 0.002] while the waiting list control group had no significant change; the group by time interaction explained 6.40% of the variance in addition to the demographic covariates that explained 9.68% of the variance. The estimated marginal means for each group at each time point as well as their variance SE are plotted in Figure 2B.

The linear mixed effects models of BDI and MAAS did not reveal any significant group by time interaction effect, although the mindfulness group had significant score increase after the intervention [*F*(1,16.86) = 4.887, *p* = 0.041].

Fixed effects in the linear mixed effects model of SCS explained 22.77% variance (Likelihood Ratio = 28.448, *p* = 0.003), and there was a significant group by time interaction effect in the model [*F*(1,32) = 11.159, *p* = 0.002] with the mindfulness group having significant score increase after the intervention [*F*(1,15.46) = 21.094, *p* < 0.001] while the waiting list control group had no significant change; the group by time interaction effect explained 11.08% of the variance in addition to the demographic covariates that explained 11.69% of the variance. The estimated marginal means for each group at each time point as well as their variance SE are plotted in Figure 2C. Linear mixed effects models of SCS subscales found significant group by time interaction effects with Self-Kindness, Isolation, and Over-Identification (*p* < 0.05), with the mindfulness group having significant score increase in Self-Kindness, Common Humanity and Mindfulness and significant decrease in Self-Judgment, Isolation and Over-Identification (*p* < 0.05), while the waiting list control group had no significant change.

### Relationships Among Variables

#### Correlations Among Questionnaire Score Changes

**FIGURE 3:**
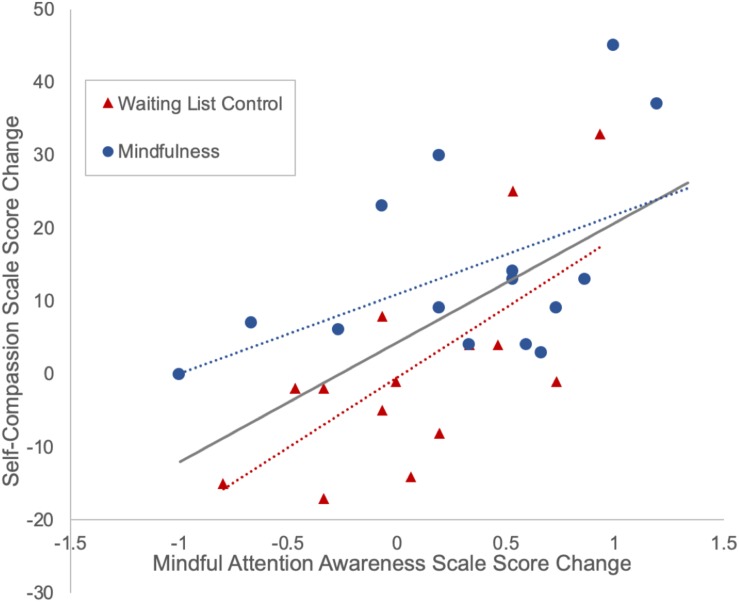
Positive correlation between changes in mindfulness and self-compassion. Subjects with more increase in mindfulness (measured by the Mindful Attention Awareness Scale) tend to have more increase in self-compassion (measured by the Self-Compassion Scale).

Partial correlation analyses showed that, with group and childhood maltreatment (as measured by MACE total severity of all types of maltreatment) as covariates, changes in mindfulness (as measured by MAAS scores) were significantly correlated with changes in self-compassion (as measured by SCS scores) among all subjects (*r* = 0.578, *p* = 0.001, df = 26). Partial correlation analyses in each group showed that after controlling for childhood maltreatment severity, changes in mindfulness and self-compassion were significantly correlated in the control group (*r* = 0.641, *p* = 0.018, df = 11) and marginally significantly correlated in the mindfulness group (*r* = 0.515, *p* = 0.059, df = 12) (Figure 3).

**FIGURE 4:**
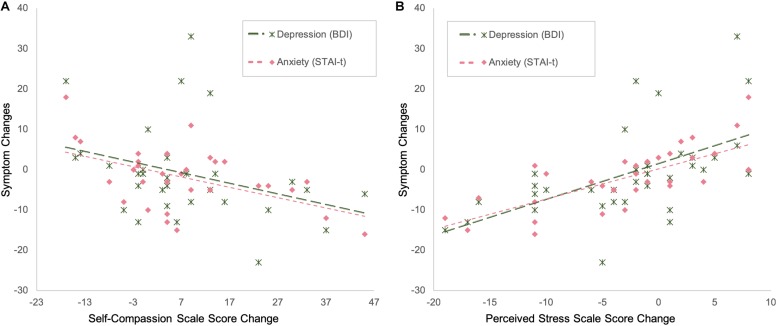
The amount of changes in depression and anxiety severity is negatively correlated with the amount of changes in self-compassion **(A)** and positively correlated with the amount of changes in stress **(B)**.

Partial correlation analyses, with group and childhood maltreatment as covariates, showed that changes in self-compassion were negatively correlated with changes in depression (SCS and BDI: *r* = −0.367, *p* = 0.05, df = 27) and anxiety (SCS and STAI-t: *r* = −0.395, *p* = 0.034, df = 27), while changes in perceived stress (as measured by PSS) were positively correlated with changes in depression (PSS and BDI: *r* = 0.591, *p* < 0.001, df = 30) and anxiety (PSS and STAI-t: *r* = 0.643, *p* < 0.001, df = 30) (Figure 4).

The correlations between the score change of SCS subscales and other questionnaires are summarized in Table 5.

**TABLE 5:** Cross correlation among score change amounts of all research questionnaires.

	**STAI-t**	**MAAS**	**PSS**	**SCS-Total**	**SCS-SK (Self-Kindness)**	**SCS-SJ (Self-Judgment)**	**SCS-CH (Common Humanity)**	**SCS-ISO (Isolation)**	**SCS-MD (Mindfulness)**	**SCS-OI (Over-identification)**
BDI	0.610^∗∗∗^	–0.270	0.591^∗∗∗^	−0.367^∗^	–0.235	0.247	–0.138	0.331	–0.099	0.286
STAI-t		–0.327	0.643^∗∗∗^	−0.395^∗^	−0.355^∗^	0.183	–0.212	0.237	–0.136	0.405^∗^
MAAS			–0.210	0.578^∗∗∗^	0.433^∗^	–0.581^∗∗∗^	0.184	−0.425^∗^	0.345	–0.233
PSS				–0.246	–0.190	0.158	–0.014	0.150	–0.016	0.400^∗^
SCS					0.744^∗∗∗^	–0.722^∗∗∗^	0.541^∗∗^	–0.753^∗∗∗^	0.518^∗∗^	–0.639^∗∗∗^
SCS_SK						–0.440^∗∗^	0.244	–0.226	0.342^∗^	–0.338
SCS_SJ							–0.050	0.538^∗∗∗^	–0.069	0.570^∗∗∗^
SCS_CH								−0.394^∗^	0.437^∗^	0.018
SCS_ISO									–0.306	0.516^∗∗^
SCS_MD										0.062

*Results are from partial correlation analyses of data from all subjects in both groups after controlling for group and childhood maltreatment severity; significance levels are indicated as: ^∗∗∗^*p* ≤ 0.001, ^∗∗^*p* ≤ 0.01, ^∗^*p* ≤ 0.05; degrees of freedom range 26–32.*

#### Mediating Effects of Self-Compassion

**FIGURE 5:**
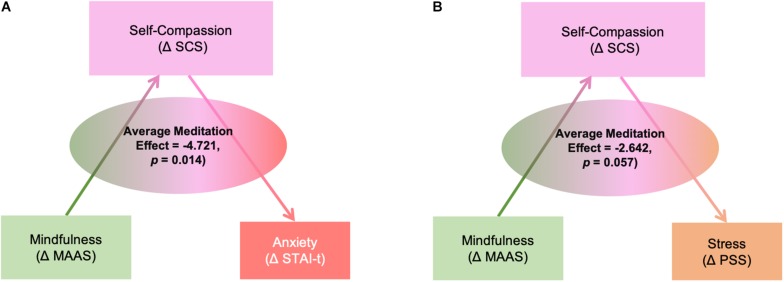
Mediation effects of self-compassion (ΔSCS: the amount of changes in scores of Self-Compassion Scale) between the changes in mindfulness (ΔMAAS: the amount of changes in scores of Mindful Attention Awareness Scale) and changes in anxiety **(A)** (ΔSTAI-t: the amount of changes in scores of State-Trait Anxiety Inventory-Trait subscale) and stress **(B)** (ΔPSS: the amount of changes in scores of Perceived Stress Scale).

Mediation analyses revealed that changes in SCS score had a significant mediating effect between MAAS and STAI-t score changes (Average Causal Mediation Effect ACME = −4.721, *p* = 0.014) (Figure 5); SCS remained a significant mediator after including group and demographic factors as covariates (ACME) = −3.337, *p* = 0.05). Mediation analyses revealed that BDI score change was not mediated by SCS. In terms of PSS, there was a marginally significant meditation effect without any covariates in the model (ACME = −2.642, *p* = 0.057), but the effect was no longer significant after adding any covariate.

#### Effects of the Amount of Class Participation and Homework Practice Compliance

**FIGURE 6:**
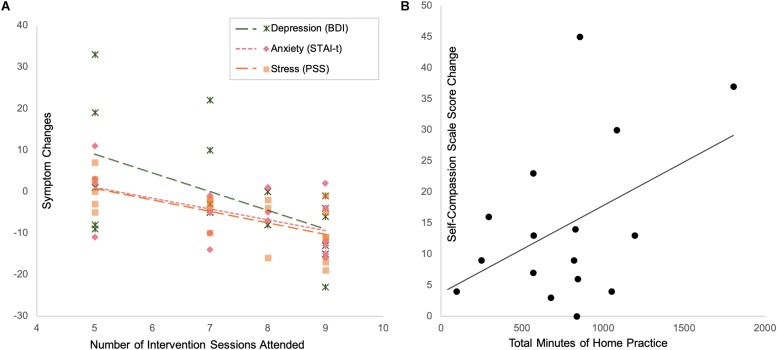
Intervention compliance and treatment effects: subjects who attended more intervention sessions had more reduction in depression, anxiety and stress **(A)**, while subjects who practiced more at home had more increase in self-compassion **(B)**.

In the mindfulness group, after controlling for the individual differences in childhood maltreatment severity, the number of intervention sessions attended was significantly associated with reduction in stress (correlation with changes in PSS: *r* = −0.674, *p* = 0.002, df = 16), anxiety (correlation with changes in STAI-t: *r* = −0.580, *p* = 0.009, df = 17), and depression (correlation with changes in BDI: *r* = −0.544, *p* = 0.020, df = 16) (Figure 6A).

With respect to the amount of homework practice, after controlling for the individual differences in childhood maltreatment severity, the reported total minutes of home practice were associated with increase in self-compassion (marginally significant correlation with changes in SCS scores: *r* = 0.468, *p* = 0.079, df = 13) (Figure 6B).

#### Effects of Childhood Maltreatment

**FIGURE 7:**
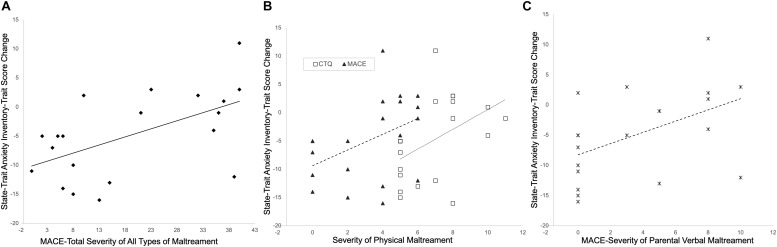
Childhood maltreatment negatively impacts treatment effects. Subjects with more severe maltreatment **(A)**, or more severe physical maltreatment as measured by Childhood Trauma Questionnaire (CTQ) or MACE **(B)**, or more severe parental verbal maltreatment **(C)**, tend to have less reduction in anxiety symptoms.

Partial correlation analyses, controlling for baseline symptom severity (pre-intervention STAI-t scores) and number of intervention sessions attended, showed that overall childhood maltreatment severity (measured by MACE-total severity scores of all types of maltreatment) negatively impacted the reduction in anxiety symptoms observed in the mindfulness group (positive correlation with changes in STAI-t scores, *r* = 0.678, *p* = 0.002, df = 16) (Figure 7A).

The specific types of maltreatment most strongly associated with changes in STAI-t scores in the mindfulness group, after controlling for pre-intervention STAI-t scores and number of intervention sessions attended, were physical abuse scores on the CTQ (*r* = 0.666, *p* = 0.003, df = 16) and ratings of parental physical maltreatment on the MACE (*r* = 0.570, *p* = 0.014, df = 16) (Figure 7B), as well as severity of parental verbal abuse on the MACE (*r* = 0.653, *p* = 0.003, df = 16) (Figure 7C).

## Discussion

This pilot study indicates that a mindfulness based behavioral intervention can be helpful for relieving stress, depression and anxiety for young adults with a childhood maltreatment history. Findings from this study also suggest that increase in self-compassion plays an important role in the therapeutic effects of the intervention. Such findings add to the literature about alternative treatments for psychological symptoms among individuals with a history of childhood maltreatment.

Previously mindfulness based interventions have been adapted and applied to treating trauma-related psychopathology such as PTSD (for review see, Banks et al., 2015; Boyd et al., 2018). The theory (Lang et al., 2012) is that mindfulness practices involves purposeful allocation of attention, which helps traumatized individuals to overcome the negative bias in their cognition and the inability to inhibit irrelevant and unwanted information (Lang et al., 2012). The “present-focus” of mindfulness practices also helps to reduce worry and rumination (Greenberg et al., 2018), which is a common contributing factor to negative affect (McLaughlin et al., 2007). The “non-judgmental” aspect of mindfulness practices helps to counteract negative interpretation of internal and external experiences (Lang et al., 2012). Although not all forms of childhood maltreatment meet the DSM definition of trauma, and only a small percentage of young adults with a childhood maltreatment history meet the DSM criteria of PTSD (Teicher et al., 2012), childhood maltreatment affects individuals’ psychological health very similarly, as mentioned above (e.g., attention bias toward negative stimuli, excessive worry and rumination, and negative interpretation of internal and external experiences). Therefore, individuals with a childhood maltreatment history may obtain similar benefits from mindfulness practices.

Previous studies about the effect of mindfulness intervention in the general population rarely report measurements of childhood maltreatment. However, childhood maltreatment is very common and thus likely introduced unknown variability in the outcome measures. One study used “Measure of Parenting Style” as a measurement of childhood experience, and found that subjects with more episodes of depression tend to have more adverse childhood experience, but they did not find significant relationship between childhood maltreatment and treatment response (Ma and Teasdale, 2004). Another study showed a mindfulness based cognitive therapy treatment was particularly helpful for protecting relapse prevention among patients with recurrent depression who had a history of childhood maltreatment (Williams et al., 2014). The present study observed increased mindfulness and reduced stress after the mindfulness based intervention, which is consistent with previous findings on the effect of mindfulness based interventions (Shapiro et al., 2005; Shapiro et al., 2007; Carmody and Baer, 2008; Baer et al., 2012a; Chin et al., 2019). The present study even observed a dose-dependent effect between the number of intervention sessions attended and symptom reduction. These findings confirm the efficacy of the mindfulness-based intervention for reducing stress in this population.

Furthermore, the present study also observed increased self-compassion after the mindfulness intervention. A previous study also found increased self-compassion after the mindfulness based stress reduction program (Birnie et al., 2010), while another study found that self-compassion is a significant predictor of psychological wellbeing among meditators (Baer et al., 2012b). Childhood maltreatment often leads to low self-esteem (Stein et al., 2002; Bungert et al., 2015) and self-criticism (Dunkley et al., 2010), which in turn contributes to other psychological health issues (Stein et al., 2002; Dunkley et al., 2010; Bungert et al., 2015). Therefore, promoting self-compassion seems to be a necessary step toward alleviating psychological symptoms influenced by childhood maltreatment. In the present study, increase in self-compassion is a significant mediator between mindfulness and symptom reduction, which is consistent with a previous study that suggests self-compassion is a better predictor than mindfulness for symptom severity of depression and anxiety among a large community sample (Van Dam et al., 2011). The present finding highlights the importance of self-compassion for treating psychological symptoms of individuals with a childhood maltreatment history.

Although self-compassion was not explicitly taught as part of the mindfulness based intervention program, self-care and self-awareness were emphasized throughout the intervention, and participants were also taught a “*loving-kindness*” meditation exercise in the later weeks of the program, which included kind wishes to people of choice, e.g., “*May you/I be happy, healthy and strong*.” These elements might have helped the cultivation of self-compassion. Furthermore, the non-judgmental and present-focused properties of mindfulness practices naturally set the stage for developing self-compassion by encouraging participants to be open and accepting of their inner experience. Future studies may consider investigating whether this population would benefit from interventions that are explicitly focused on improving self-compassion, such as the mindful self-compassion program developed by Neff and Germer (2013).

The severity of childhood maltreatment appeared to have negatively impacted the efficacy of the intervention. Individuals with more severe childhood maltreatment had less reduction in anxiety symptoms, especially individuals with more severe parental physical or verbal maltreatment. Our finding is consistent with the existing literature that individuals with a childhood maltreatment history are difficult to treat (e.g., Shamseddeen et al., 2011; Harkness et al., 2012; Tunnard et al., 2014). Such results bring up the possibility that this mindfulness-based program might have limited benefits for individuals with severe childhood maltreatment, who might require a longer program or multiple treatment approaches, such as a combination of individual psychotherapy, group therapy, mindfulness practice and explicit self-compassion training.

There are several limitations in the present study, including (1) a relatively small sample, which limits the generalizability of the findings; (2) a waiting list control instead of an active control condition, which could have been confounded by placebo effects; (3) subjects were not randomized, but were rather assigned based on their availability to attend the intervention sessions, which could have resulted in the two groups being different, e.g., subjects on the waiting list group might have busier schedules. Although the two groups had no significant difference on their baseline stress levels or any of the psychological instruments used in the present study, they could have been different on some unmeasured factors. Therefore, strict randomization procedures shall be applied on future studies. (4) subjects were not followed up with after they finished the program to observe the long-term effect of the intervention; future studies that include long term follow up assessments can help understand how long the benefits from the intervention can last. (5) subjects were predominantly female, future studies should recruit more male participants. (6) lack of comprehensive information about subjects’ personality and social support system limit our understanding about potential societal factors that could contribute to treatment effects. (7) stress is a multifaceted concept, we only measured subjects’ subjective report of perceived stress, more comprehensive measurement that includes biological markers of stress could reveal more information about the treatment’s effect on stress response. (8) most subjects in this study only had mild to moderate psychological symptoms, which limits the generalizability of the study to more symptomatic patients.

This pilot study provided promising initial findings that young adults with a childhood trauma history can benefit from a mindfulness-based intervention. Given that this population tends to be resistant to traditional treatments, mindfulness-based interventions are worth considering as one of the alternative treatment approaches for psychological symptoms related to childhood maltreatment.

## Data Availability Statement

The datasets generated for this study are available on request to the corresponding author.

## Ethics Statement

This study was reviewed and approved by the Institutional Review Board (IRB) (IRB#: 2014P000295) of Partners HealthCare, which is the IRB for Massachusetts General Hospital, McLean Hospital, and several other major hospitals in the Boston area. The patients/participants provided their written informed consent to participate in this study.

## Author Contributions

DJ and SL planned the experiments and supervised the intervention programs. AK performed clinical screening for all subjects. MT provided subject database with all childhood maltreatment assessments. DJ conducted the experiments and data analyses and drafted the manuscript. All authors contributed to the final version of the manuscript.

## Conflict of Interest

The authors declare that the research was conducted in the absence of any commercial or financial relationships that could be construed as a potential conflict of interest.
